# Oxidative Stress Biomarkers and Their Association with Mortality among Patients Infected with SARS-CoV-2 in Mexico

**DOI:** 10.1155/2022/1058813

**Published:** 2022-06-17

**Authors:** Azalia Avila-Nava, Alfredo Geovanny Pech-Aguilar, Roberto Lugo, Isabel Medina-Vera, Martha Guevara-Cruz, Ana Ligia Gutiérrez-Solis

**Affiliations:** ^1^Hospital Regional de Alta Especialidad de la Península de Yucatán (HRAEPY), Mérida, 97130 Yucatán, Mexico; ^2^Becario de la Dirección General de Calidad y Educación en Salud (DGCES), Secretaria de Salud, Ciudad de Mexico, Mexico; ^3^Departamento de Metodología de la Investigación, Instituto Nacional de Pediatría (INP), Ciudad de Mexico 04530, Mexico; ^4^Departamento de Fisiología de la Nutrición, Instituto Nacional de Nutrición y Ciencias Médicas Salvador Zubirán (INCMNSZ), Ciudad de Mexico 14080, Mexico

## Abstract

**Background:**

Activation of the immune system response is associated with the generation of oxidative stress (OS). Several alterations are involved in OS, such as excessive production of reactive oxygen species (ROS) and decreased antioxidant activity, which together lead to an imbalance in redox status. The role of OS during SARS-CoV-2 infection is not fully understood. The aim of this study was to determine OS biomarkers and assess their usefulness as a predictor of mortality in COVID-19 patients.

**Methods:**

Baseline characteristics and serum samples were collected from hospitalized COVID-19 patients and compared with healthy controls. The serum OS biomarkers, including malondialdehyde (MDA) and total antioxidant capacity (TAC), were assessed by spectrophotometric and oxygen radical absorbance capacity (ORAC) methods, respectively.

**Results:**

A total of 152 individuals were analyzed (COVID-19 patients vs. healthy controls). Compared with healthy controls (*n* = 76), patients infected with SARS-CoV-2 (*n* = 76) presented higher levels of MDA (*p* < 0.001) and decreased TAC (*p* < 0.001). A total of 37 (49%) patients with COVID-19 died. The area under the receiver operating characteristic (ROC) curve (AUC) estimated that the combination of the OS biomarkers (MDA+TAC) (AUC = 0.6394, *p* = 0.037) was a significant predictor of mortality. A higher level of MDA was associated with mortality (HR, 1.05, 95% CI, 1.00–1.10, *p* = 0.045).

**Conclusion:**

This study concludes that OS is increased in patients with COVID-19 and is associated with mortality. To our knowledge, this is the first evidence of the expression of OS biomarkers and their association with mortality among the Mexican population.

## 1. Introduction

The novel coronavirus disease 2019 (COVID-19) is caused by severe acute respiratory syndrome coronavirus 2 (SARS-CoV-2) and has become a major public health problem worldwide, leading to 5,551,314 deaths as of January 2022 [1]. Some major risk factors for developing severe COVID-19 include advanced age, increased body mass index (BMI), type 2 diabetes (T2D), and hypertension, among others [2]. Additionally, it has been reported that among individuals with worse COVID-19 outcomes, inefficient immune responses promote hyperinflammation, leading to a cytokine storm [3, 4]. Furthermore, activation of immune system responses is associated with the generation of oxidative stress (OS) [5]. Several alterations are involved in OS, such as excessive production of reactive oxygen species (ROS) and decreased antioxidant activity, which lead to an imbalance in the redox status [6]. Altogether, these changes trigger oxidative damage that has been linked to tissue injury by the oxidation of different biomolecules, including nucleic acids, proteins, and lipids, which also play an important role in the complex pathogenesis of COVID-19 [7–9].

During lipid peroxidation (oxidation of lipids by ROS), excessive compounds are generated, such as the oxidative marker malondialdehyde (MDA). These types of compounds can act as cytotoxic products, modifying the structures and functions of different cell components [10, 11].

The role of OS during SARS-CoV-2 infection is not fully understood. However, in some animal and human studies, it has been reported that other viral infections, such as human immunodeficiency virus (HIV) [12] and hepatitis C virus (HCV) [13], among others [14], trigger the production of ROS, promoting metabolic dysfunction [15].

Although hyperinflammation and OS are closely related, the vast majority of the literature have focused on the cytokine storm with very few studies aimed at elucidating the status of OS in COVID-19 patients. Moreover, it has been well described that elevation of some serum inflammation biomarkers, such as interleukin-6 (IL-6) [16] and necrosis factor alpha (TNF-*α*) [17], among others [3], is associated with mortality in COVID-19 patients. Considering these alterations may be associated with severe damage to the respiratory system and the malfunctions of various organs, together they may contribute to fatal outcomes in infected patients. Despite the theoretical knowledge of the role of OS in the evolution of COVID-19, there is still a lack of further studies on biomarkers of the redox status in patients. Therefore, we hypothesized that COVID-19 cases had an increase in OS and that this may be associated with mortality. Evidence can then be used to generate strategies to prevent the development and progression of severe damage by COVID-19. Against this background, the aim of the present study was to determine OS and assess its usefulness as a predictor of mortality among COVID-19 patients.

## 2. Materials and Methods

### 2.1. Study Design

This study used data from two cross-sectional studies that are aimed at identifying serum levels of inflammatory biomarkers in hospitalized patients with COVID-19 at the Regional High Specialty Hospital (HRAEPY in the Spanish acronym) in Merida, Yucatan, Mexico, during the first wave of the COVID-19 pandemic between April and December 2020. Patients with COVID-19 are classified as severe and critical disease according to the clinical management of COVID-19 by the WHO [18]. Patients with critical disease are those who require mechanical ventilation, sedation, and prolonged bed rest. Participants recruited were not vaccinated against SARS-CoV-2 infection. The sample size was calculated using a simple random formula for an unknown population [19]. The parameters were 90% confidence, and 10% estimation error. From this calculation, the minimum required sample size was 68 patients. A total of 76 patients with COVID-19 were included. The study was approved by the Ethics Committee of our hospital (No. CONBIOETICA31-CEI-002-20170731) in connection with 2 research projects (Identification codes: 2020-010 and 2020-021). Healthy controls were part of a cross-sectional study performed in 2018, also approved by the Ethics Committee of our hospital (Identification code: 2017-025).

#### 2.1.1. Subjects: Inclusion and Exclusion Criteria

The following inclusion criteria were applied: patients with confirmed SARS-CoV-2 infection by polymerase chain reaction (PCR) performed within 72 hours after hospital admission, patients with severe and critical disease classified according to the clinical management of COVID-19 by the WHO [18], and patients aged 18 years or older. No exclusion criteria were applied for patients with COVID-19.

For the control group, serum samples from 76 (56 males and 20 females) healthy subjects without SARS-CoV-2 infection who were age- and sex-matched were used as controls.

### 2.2. Data Collection

Baseline characteristics, such as demographics, clinical data, and serum samples, were collected within the first 72 hours after hospital admission into the COVID unit. Data from participants, including age, sex, blood pressure, and comorbidities, such as T2D, hypertension, and weight status based on BMI classification, were categorized. Additionally, serum samples were used to determine biochemical parameters, including fasting plasma glucose (FPG), urea, creatinine, uric acid (UA), ferritin, and C-reactive protein (CRP), using prevalidated equipment (autoanalyzer COBAS® Integra 400 Plus, Roche Diagnostics, Mannheim, Germany).

### 2.3. Measurement of Redox Status by Oxidative and Antioxidant Biomarkers

Serum antioxidant capacity was defined as a decrease in total antioxidant capacity (TAC) and an increase in MDA levels. Therefore, TAC was evaluated by the oxygen radical absorbance capacity (ORAC) assay [20]. Briefly, the assay was performed using the following reaction: 25 *μ*L of serum (1 : 100), 25 *μ*L of 2,2-azobis(2-amidinopropane) dihydrochloride (153 mM), and 150 *μ*L of fluorescein (4 mM). The fluorescence signal was measured at 485 nm (excitation) and 535 nm (emission) at 1 min intervals for 90 min in a Synergy HT plate reader (BioTek Instruments, Winooski, VT). All data on antioxidant capacity were calculated using Trolox as a standard and expressed as micromoles of Trolox equivalents (TE) per milliliter of sample. Furthermore, the serum concentration of MDA was determined by a spectrophotometric method, quantified at a wavelength of 586 nm, and reported as nanomoles per milliliter [21].

### 2.4. Statistics

The statistical package Jamovi (version 2.25, Sydney, Australia) was used to analyze the data. Baseline demographic, clinical, and biochemical characteristics are presented as the means ± standard deviation (SD), and in the case of nonnormal distributions, medians (interquartile range [IQR]) are reported, and comorbidities are described using proportions with corresponding percentages (%). Evaluation of normality was performed using the Shapiro–Wilk normality test. Continuous variables between groups were compared using Student's *t*-test for independent samples or the Mann–Whitney *U* test. The area under the receiver operating characteristic (ROC) curve (AUC) was estimated using binomial logistic regression analysis, which was used to assess the discriminatory abilities of MDA and TAC to predict mortality (yes = 1; no = 0). The survival analysis was assessed using the Kaplan–Meier survival function curve to test statistical significance between the individuals who had a high level of MDA and TAC. A *p* value ≤ 0.05 was considered statistically significant.

## 3. Results

A total of 152 individuals were analyzed (COVID-19 patients vs. healthy controls), including 76 (56 males and 20 females) patients admitted to the COVID unit aged between 21 and 79 years and 76 matched controls. Patients with severe (*n* = 33) and critical (*n* = 43) disease were compared, but no significant differences in clinical parameters were found between the groups in Supplementary Table S1. COVID-19 patients (*n* = 76) were significantly older (53.6 ± 14.1; *p* = 0.001) and had higher rates of comorbidities such as obesity (66%), hypertension (38%), and T2D (32%) (Table 1). Additionally, patients with COVID-19 had significantly higher levels of systolic blood pressure (SBP) (*p* = 0.020), diastolic blood pressure (DBP) (*p* = 0.001), FPG (*p* < 0.001), urea (*p* = 0.003), ferritin (*p* < 0.001), CRP (*p* < 0.001), and MDA (*p* = 0.038) than healthy controls. However, healthy controls had higher serum levels of creatinine (*p* = 0.001), UA (*p* < 0.001), and TAC (*p* < 0.001) (Table 2). No differences in OS biomarkers between males and females were found in Supplementary Table S2.

**Figure 1:**
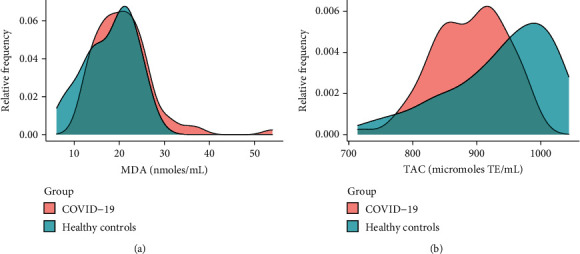
Estimation of the distributions using kernel density of serum (a) malondialdehyde (MDA) and (b) total antioxidant capacity (TAC) levels among subjects with COVID-19 (*n* = 76) and healthy controls (*n* = 76).

Increased serum levels of MDA (20.5 [16.8, 24.2] vs. 19.1 [14, 22.1] nmoles/mL, *p* = 0.038) and lower TAC levels (899 [847, 929] vs. 956 [833, 1006] micromoles of TE/mL, *p* < 0.001) were found in patients with COVID-19 versus healthy controls (Figure 1). For MDA, a cutoff value of 25.15 nmoles/mL with a sensitivity of 43% and specificity of 61% with a Youden Index of 0.22 was established. For TAC, a cutoff value of 882.9 micromoles of TE/mL with a sensitivity of 68% and specificity of 64% with a Youden Index of 0.34 was established.

**Figure 2:**
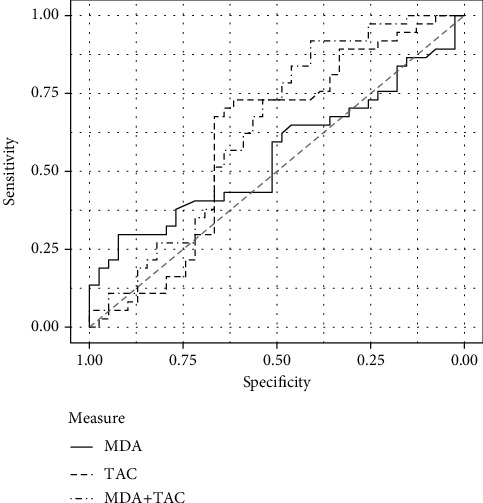
Area under the receiver operating characteristics curve of malondialdehyde (MDA) and total antioxidant capacity (TAC) to predict mortality in COVID-19 patients (*n* = 76).

A total of 37 (49%) patients with COVID-19 died, and they were significantly older (60.6 ± 11.5 years) than patients who survived (47.3 ± 11.45 years). Using binomial regression, the AUCs of some variables were assessed to predict mortality among patients infected with SARS-CoV-2. Age and serum levels of CRP, MDA, and TAC and the combination of the OS biomarkers (MDA+TAC) were evaluated. The AUCs of age (AUC = 0.766, *p* < 0.001), CRP (AUC = 0.638, *p* = 0.039), and MDA+TAC (AUC = 0.639, *p* = 0.037) were greater and significant, suggesting their significance as predictors of mortality (Table 3, Figure 2). The AUCs are also shown in Figure 2.

**Figure 3:**
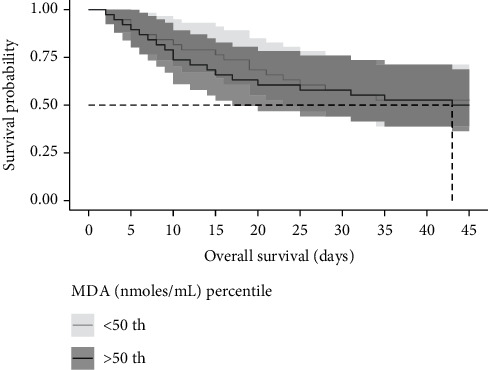
Kaplan–Meier curve according to malondialdehyde (MDA) levels above the 50th percentile in COVID-19 patients. The cumulative mortality at a time to event (nonsurvivor or survivor).

**Figure 4:**
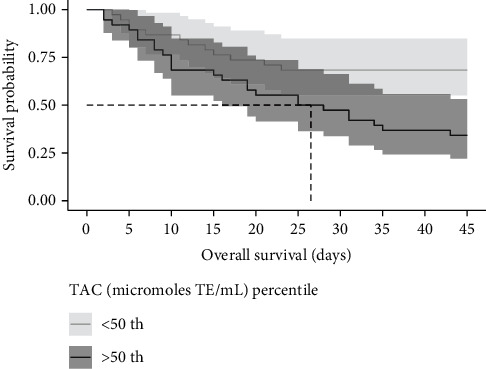
Kaplan–Meier curve according to total antioxidant capacity (TAC) levels above the 50th percentile in COVID-19 patients. The cumulative mortality at a time to event (nonsurvivor or survivor).

The probability of death given MDA and TAC levels was calculated using survival analyses with a 50% cutoff. Individuals who were above the 50% percentile in MDA levels (19.5 nmol/mL) died within a median of 43 days (Figure 3). Half of the individuals who were above the 50% percentile in TAC levels (921 micromoles of TE/mL) died within a median of 26.5 days (Figure 4).

In addition, a Cox model estimated that the likelihood of death was associated with age (HR, 1.05, 95% CI, 1.02–1.08, *p* < 0.001) and increased levels of MDA (HR, 1.05, 95% CI, 1.00–1.10, *p* = 0.045). This suggests that for every year of age, the probability of mortality increased by 5%, and for each 100 units of nmoles/mL, the probability of mortality increased. Although age had a stronger association with mortality, MDA also showed a significant positive association. This alteration in MDA levels suggests an increase in OS in deceased patients (Table 4).

## 4. Discussion

In our study, it was found that serum levels of MDA showed a significant increase in deceased COVID-19 patients, suggesting that the OS status among these patients was higher than healthy controls and COVID-19 survivors. Moreover, TAC levels were significantly lower in subjects infected with COVID-19 than that in healthy controls. The combination of these two markers (MDA+TAC) was shown to be a good predictor of mortality among COVID-19 patients.

Measuring biomarkers of OS in subjects from clinical serum samples can be challenging due to their short half-lives [22], especially for TAC [23]. MDA and TAC have been shown to predict worse clinical outcomes in previous studies with lifestyle-related diseases, including patients with T2D [24], metabolic syndrome [25], and cardiovascular disease [26], but only a few reports have been reported on patients with COVID-19.

The rate of mortality in the present study sample was very high (49%), which was in accordance with other reports that indicated Mexico is one of the countries with the highest mortality rates [27]. Moreover, individuals who did not survive were older (60.6 ± 11.5 years) than patients who survived (47.3 ± 11.45 years). Age is one of the main determinants of mortality in patients with COVID-19 [28]. In a recent study, it was found that higher age is the greatest contributor to mortality in hospitalized patients with COVID-19 in the Netherlands. In fact, the mediation effect of preexisting comorbidities (hypertension, T2D, dyslipidemia, etc.) in mortality is minimal [29].

However, very few studies have explored the levels of some biomarkers for OS among COVID-19 patients. In a pilot study by Pincemail et al. including nine hospitalized patients from Belgium, they found increased lipid peroxidation and deficits in some antioxidants (vitamin C, glutathione, and thiol proteins) and trace elements (selenium). This suggests an alteration in the systemic redox status [8]. In another retrospective study in France by Schmitt et al. that enrolled 15 pregnant women with and without COVID-19 during their third trimester, no differences were shown between CRP levels and OS markers between groups [30]. Another report from Iran by Karkhanei et al. including 96 individuals with and without COVID-19 found that elevation in serum levels of OS (reduced glutathione and TAC) and reduction of antioxidant indices (total oxidant status) could aggravate the disease's severity in hospitalized patients with COVID-19. However, levels of TAC were not significant when compared with non-ICU patients [31]. There are no records or studies in the Mexican population on the relationship between serum levels of OS biomarkers and mortality in SARS-CoV-2-infected patients. In our study, hospitalized patients with COVID-19 from Mexico showed elevated levels of MDA and reduced levels of antioxidant capacity. Nevertheless, deceased patients with COVID-19 had high levels of TAC. This finding could be explained by the fact that, given the excessive increase in ROS, the antioxidant system is unable to mitigate the generation of OS. Therefore, the oxidative damage would be irreversible in patients who did not survive. Another study's findings reported an imbalance between ROS levels and antioxidants during the evolution of COVID-19. In fact, patients from Serbia with moderate COVID-19 showed lower activity of the antioxidant enzyme catalase than individuals with severe disease, who also reported higher levels of superoxide anion radicals [32]. Excessive amounts of ROS can initiate an intracellular signaling cascade to induce the activation of transcriptional nuclear factor B (NF-*κ*B), which promotes the overexpression of proinflammatory genes [33]. However, data from our study included hospitalized patients who developed severe and critical disease, but no differences in the clinical, OS, and inflammatory biomarkers were found when these two groups are compared in Supplementary Table S1. Moreover, serum levels of MDA and TAC were not shown to have a significant relationship with CRP and/or ferritin levels in COVID-19 patients in Supplementary Table S3. These results can be attributed to the fact that most hospitalized patients showed severe oxidative damage due to COVID-19 infection.

Among inflammatory markers, IL-6 and CRP have been postulated to be good biomarkers in the prediction of mortality. In fact, in a prospective meta-analysis of clinical trials of patients hospitalized for COVID-19, it was concluded that administration of IL-6 antagonists was associated with lower all-cause mortality with a cutoff of 28 days [34]. Considering the close relationship between hyperinflammation and OS, it may be deduced that antioxidants could be an alternative to reduce the OS status in COVID-19 patients. To date, only one study protocol for a randomized controlled trial has been registered to explore the efficacy of cosupplementation with curcumin-piperine on OS factors and mortality in patients with COVID-19 admitted to the intensive care unit (ICU) in Iran; however, no published results have been found [35]. On the other hand, the beneficial effects of some natural bioactive compounds during COVID-19 infection are understudied. A study by Shohan et al. showed that intervention with combined quercetin (1000 mg/day), remdesivir, and favipiravir promoted a decrease in serum levels of CRP and lactate dehydrogenase (LDH) [36]. Another study in Iran pointed out that supplementation with vitamin C (500 mg/day) increased the survival duration in critical COVID-19 patients compared with the control group (8 vs. 4 days, *p* < 0.01) [37].

These natural compounds have various biological functions related to them, including antioxidant and anti-inflammatory properties [38, 39]. Some of the beneficial effects could be associated with their capacity to neutralize excessive ROS levels and activate the nuclear antioxidant erythroid factor 2-related factor 2 (Nrf2) pathway [40]. Additionally, natural compounds can also modulate the inflammatory pathway by inhibiting NF-*κ*B [41]. In this context, more research that elucidates the role of bioactive compounds against OS and inflammation as a therapeutic alternative for patients with COVID-19 is needed.

Some limitations of this study are the short half-lives of TAC. Nevertheless, some studies report the measurement of this biomarker from serum samples [42–44]. Moreover, MDA is an end product of lipid peroxidation; thus, it is stable and has a long half-life. Although serum samples from nonhospitalized patients with mild and moderate disease were not included in our study, healthy controls (age- and sex-matched) were used to compare the presence of OS. The strengths of our study include that the data presented correspond to the first wave of COVID-19 and a relatively large number of patients were included in the analyzed groups. Remarkably, to our knowledge, this is the first evidence of the expression of OS biomarkers and their association with mortality among the Mexican population.

## 5. Conclusions

Together, elevated levels of MDA and decreased levels of TAC are found among COVID-19 patients, and the combination of both biomarkers is a predictor of mortality among these patients. Moreover, an elevation in MDA levels may increase the probability of mortality due to COVID-19 in patients from Mexico. Further research in patients infected with SARS-CoV-2 to elucidate the molecular mechanisms and the interaction between these OS biomarkers and other mediators is needed.

## Figures and Tables

**Table 1:** Baseline demographic and comorbidities of subjects with COVID-19 and matched healthy controls.

Characteristics	COVID-19 (*n* = 76)	Healthy controls (*n* = 76)	*p* value
Age (y), mean (SD)	53.6 (14.10)	45.6 (13.30)	0.001
Male, *n* (%)	56 (74)	56 (74)	1.00
Comorbidities			
T2D, *n* (%)	24 (32%)	11 (15%)	0.013
Hypertension, *n* (%)	29 (38%)	19 (25%)	0.08
Obesity, *n* (%)	50 (66%)	56 (74%)	0.92

T2D: type 2 diabetes. *p* value less than 0.05 was considered statistically significant.

**Table 2:** Clinical characteristics of subjects with COVID-19 and matched healthy controls.

Characteristics	COVID-19 (*n* = 76)	Healthy controls (*n* = 76)	*p* value
SBP (mmHg)	125 (16.9)	118 (18)	0.020
DBP (mmHg)	75.9 (12.5)	69.8 (10)	0.001
FPG (mg/dL)	139 [101, 176]	91.7 [86.3, 97]	<0.001
Urea (mg/dL)	35 [24.5, 48.4]	24.6 [20.8, 36.4]	0.003
Creatinine (mg/dL)	0.80 [0.66, 0.95]	0.90 [0.76, 1.10]	0.001
UA (mg/dL)	3.85 [3.20, 4.80]	5.50 [4.50, 6.53]	<0.001
Ferritin (ng/mL)	1290 [830, 1973]	203 [121, 289]	<0.001
CRP (mg/dL)	197 [89, 273]	2.13 [1.35, 10]	<0.001
MDA (nmoles/mL)	20.5 [16.8, 24.2]	19.1 [14, 22.10]	0.038
TAC (micromoles TE/mL)	889 [847, 929]	956 [833, 1006]	<0.001

Data is presented using mean (standard deviation) or median [interquartile range]. Differences between groups were evaluated with independent *t*-test or Mann–Whitney test. *p* value less than 0.05 was considered statistically significant. SBP: systolic blood pressure; DBP: diastolic blood pressure; FPG: fasting plasma glucose; UA: acid uric; CRP: C-reactive protein; MDA: malondialdehyde; TAC: total antioxidant capacity.

**Table 3:** Area under the receiver operating characteristic curve of OS biomarkers and others parameters in nonsurvivor COVID-19 patients (*n* = 76).

Predictors	AUC (95% CI)	*p* value	HR AUC (95% CI)
Age	0.766 (0.660-0.872)	<0 .001	1.08 (1.04-1.12)
CRP	0.638 (0.512-0.764)	0.039	1.004 (1.00-1.01)
MDA	0.553 (0.421-0.685)	0.427	1.06 (0.98-1.14)
TAC	0.601 (0.469-0.733)	0.129	1.01 (0.99-1.02)
MDA+TAC	0.639 (0.513-0.765)	0.037	1.04 (1.01-1.08)

Dependent variable: mortality (yes = 1, no = 0). *p* value less than 0.05 was considered statistically significant. AUC: area under curve; 95% CI: 95% confidence interval; HR: hazard ratio; CRP: C-reactive protein; MDA: malondialdehyde; TAC: total antioxidant capacity.

**Table 4:** Maximum likelihood for mortality prediction among COVID-19 patients.

Parameter	Hazard ratio	95% CI	*p* value
Age (years)	1.05	1.02-1.08	<0.001
Hypertension	1.33	0.70-2.56	0.385
MDA	1.05	1.00-1.10	0.045
TAC	1.00	0.99-1.01	0.162

MDA: malondialdehyde; TAC: total antioxidant capacity; 95% CI: 95% confidence interval. *p* value less than 0.05.

## Data Availability

The datasets analyzed during the current study and the supplementary material files are available from the corresponding author on reasonable request.

## Supplementary Materials

Supplementary Materials Table S1: clinical characteristics of subjects with COVID-19 (*n* = 76) clasified into severe and critical disease. Table S2: clinical characteristics of subjects with COVID-19 (*n* = 76) and healthy controls (*n* = 76) classified by sex. Table S3: correlations between inflammatory markers with oxidative stress biomarkers among patients with COVID-19 (*n* = 76) and healthy controls (*n* = 76).
