# Supplementary material for: Oxidative Stress Biomarkers and Their Association with Mortality among Patients Infected with SARS-CoV-2 in Mexico
Source: Oxid Med Cell Longev. 2022 Jun 17;2022:1058813. doi: 10.1155/2022/1058813 (PMC9210126; doi:10.1155/2022/1058813)
Supplement: Supplementary Materials — Table S1: clinical characteristics of subjects with COVID-19 (n = 76) clasified into severe and critical disease. Table S2: clinical characteristics of subjects with COVID-19 (n = 76) and healthy controls (n = 76) classified by sex. Table S3: correlations between inflammatory markers with oxidative stress biomarkers among patients with COVID-19 (n = 76) and healthy controls (n = 76). [file 1058813.f1.docx]

**SUPPLEMENTARY MATERIAL**

**Supplementary Table S1.** Clinical characteristics of subjects with COVID-19 (n=76) clasified into severe and critical disease.

| **Characteristics** | **Severe**  **(n=33)** | **Critical**  **(n=43)** | ***p-*value** |
| --- | --- | --- | --- |
| SBP (mmHg) | 123 (18.5) | 126 (15.15) | 0.322 |
| DBP (mmHg) | 74.7 (14.4) | 76.8 (10.6) | 0.469 |
| FPG (mg/dL) | 138 [96.3, 176] | 140 [109, 174] | 0.664 |
| Urea (mg/dL) | 27.9 [20.6, 44] | 37.6 [28.1, 51.5] | 0.062 |
| Creatinine (mg/dL) | 0.79 (0.16) | 0.81 (0.24) | 0.752 |
| UA (mg/dL) | 3.92 (1.47) | 4.13 (1.63) | 0.576 |
| Ferritin (ng/mL) | 1552 [1037, 1962] | 933 [685, 2087] | 0.081 |
| CRP (mg/dL) | 198 (106) | 194 (127) | 0.895 |
| MDA (nmoles/mL) | 21 [17, 23.7] | 19.9 [19.9, 24.5] | 0.564 |
| TAC (micromoles TE/mL) | 883 (60.2) | 894 (54) | 0.444 |

Data is presented using mean (Standar deviation) or median [Interquartile range]. Differences between groups were evaluated with independent t-test or Mann-Whitney test. p-value less than 0.05 was considered statistically significant.

SBP: Systolic blood pressure; DBP: Diastolic blood pressure; FPG: Fasting plasma glucose; UA: Acid uric; CRP: C-reactive protein, MDA: Malondialdehyde; TAC: Total antioxidant capacity.

**Supplementary Table S2.** Clinical characteristics of subjects with COVID-19 (n=76) and healthy controls (n=76) classified by sex.

| **COVID-19 Healthy Controls** | | | | | | |
| --- | --- | --- | --- | --- | --- | --- |
| **Characteristics** | **Men**  **(n=56)** | **Women**  **(n=20)** | ***p-*value** | **Men**  **(n=56)** | **Women**  **(n=20)** | ***p-*value** |
| SBP (mmHg) | 125.67 (16.7) | 122.25 (17.48) | 0.439 | 121 (18.8) | 110 (12.8) | 0.017 |
| DBP (mmHg) | 77.42 (11.5) | 71.7 (13.96) | 0.078 | 70.9 (103) | 69 (8.84) | 0.133 |
| FPG (mg/dL) | 142 [11, 176] | 124 [94.5, 201] | 0.498 | 91.8 [86.7, 97] | 94 [83.6, 97.5] | 0.706 |
| Urea (mg/dL) | 32.8 [25.6, 51] | 37.6 [16.9, 43.7] | 0.280 | 26.6 [21.5, 37.3] | 23.6 [15.6, 29.9] | 0.077 |
| Creatinine (mg/dL) | 0.83 (0.2) | 0.73 (0.22) | 0.083 | 0.94 [0.88, 1.15] | 0.73 [0.62, 0.73] | <0.001 |
| UA (mg/dL) | 4.1 (1.6) | 3.8 (1.4) | 0.415 | 6.10 (5.1, 6.8) | 4.3 [3.53, 5.03] | <0.001 |
| Ferritin (ng/mL) | 1377 [916, 2132] | 900 [479, 1490] | 0.019 | 239 [142, 334] | 106 [46.1, 185] | <0.001 |
| CRP (mg/dL) | 204 (116.3) | 171 (122) | 0.278 | 2.12 [1.5, 7.52] | 2.14 [1.29, 19.35] | 0.850 |
| MDA (nmoles/mL) | 2.1 [16, 23.1] | 20.9 [17.6, 25.4] | 0.190 | 17.7 (6.01) | 19.1 (4.22) | 0.344 |
| TAC (micromoles TE/mL) | 889 (58) | 889.1 (54.4) | 0.996 | 952 [883, 1006] | 967 [889, 1004] | 0.71 |

Data is presented using mean (Standar deviation) or median [Interquartile range]. Differences between groups were evaluated with independent t-test or Mann-Whitney test. p-value less than 0.05 was considered statistically significant.

SBP: Systolic blood pressure; DBP: Diastolic blood pressure; FPG: Fasting plasma glucose; UA: Acid uric; CRP: C-reactive protein, MDA: Malondialdehyde; TAC: Total antioxidant capacity.

**Supplementary Table S3.** Correlations between inflammatory markers with oxidative stress biomarkers among patients with COVID-19 (n = 76) and Healthy controls (n = 76).

| **COVID-19**  **Inflammatory markers** | | | | **Healthy Controls**  **Inflammatory markers** | | | |  |
| --- | --- | --- | --- | --- | --- | --- | --- | --- |
|  | **CRP** | | **Ferritin** | | **CRP** | | **Ferritin** | |
| **Oxidative**  **biomarkers** | ***r*** | ***p*- value** | ***r*** | ***p*-value** | ***r*** | ***p*-value** | ***r*** | ***p*-value** |
| MDA | 0.004 | 0.487 | -0.005 | 0.584 | 0.036 | 0.378 | 0.199 | 0.042 |
| TAC | -0.008 | 0.473 | -0.017 | 0.813 | 0.107 | 0.822 | -0.027 | 0.407 |

CRP: C-reactive protein; MDA: Malondialdehyde; TAC: Total antioxidant capacity. p-value less than 0.05 was considered statistically significant.
